# Analysis of the characteristics of slot design affecting resistance to sliding during active archwire configurations

**DOI:** 10.1186/2196-1042-14-35

**Published:** 2013-10-01

**Authors:** Riccardo Nucera, Antonino Lo Giudice, Giovanni Matarese, Alessandro Artemisia, Ennio Bramanti, Paolo Crupi, Giancarlo Cordasco

**Affiliations:** 1 Department of Scienze Sperimentali Medico-Chirurgiche ed Odontostomatologiche, Section of Orthodontics and TMJ Disorder, University of Messina, Via Consolare Valeria, Messina, 98123, Italy; 2 Department of Scienze Sperimentali Medico-Chirurgiche ed Odontostomatologiche”, Section of Oral Surgery, University of Messina, Via Consolare Valeria, Messina, 98123, Italy

**Keywords:** Bracket design, Resistance to slide, Self-ligating

## Abstract

**Background:**

During orthodontic treatment, a low resistance to slide (RS) is desirable when sliding mechanics are used. Many studies showed that several variables affect the RS at the bracket-wire interface; among these, the design of the bracket slot has not been deeply investigated yet. This study aimed to clarify the effect of different slot designs on the RS expressed by five types of low-friction brackets in vertical and horizontal active configurations of the wire.

**Methods:**

Five low-friction brackets (Damon SL II, Ormco, Orange, CA, USA; In-Ovation, GAC International, Bohemia, NY, USA; Quick, Forestadent, Pforzheim, Germany; Time 2, AO, Sheboygan, WI, USA; Synergy, RMO, Denver, CO, USA) coupled with an 0.014-in NiTi thermal wire (Therma-Lite, AO) were tested in two three-bracket experimental models simulating vertical and horizontal bracket displacements. A custom-made machine was used to measure frictional resistance with tests repeated on ten occasions for each bracket-wire combination. Design characteristics such as the mesio-distal slot width, slot depth, and presence of chamfered edges at the extremities of the slot were evaluated on SEM images (SUPRA, Carl Zeiss, Oberkochen, Germany) and analyzed in relation to the data of RS recorded.

**Results:**

Time 2 was found to show the higher frictional forces (1.50 and 1.35 N) in both experimental models (*p* < 0.05), while Quick and Synergy brackets showed the lower frictional values in the vertical (0.66 N) and in the horizontal (0.68 N) bracket displacements, respectively. With vertically displaced brackets, the increased mesio-distal slot width and the presence of clear angle at mesial and distal slot edges increase the values of RS. With brackets horizontally displaced, the RS expressed by the wire is influenced simultaneously by the depth of the slot, the mesio-distal slot width, and the presence of clear angle at the extremities of the slot base, the clip, or the slide.

**Conclusion:**

In order to select the proper low-friction bracket system, clinicians should consider specific characteristics of slot design apart from the wire engaging method.

## Background

In the modern straight-wire mechanics, the sliding of the wire through brackets and tubes is fundamental in achieving the alignment of the dental arch [1]. A range from 12% to 60% of the orthodontic forces applied is expected to be lost due to resistance to sliding (RS) [2], reducing the amount of forces exerted by fixed appliance. This wide range of variability reduces the predictability of the applied forces; for this reason, a better understanding of the RS is mandatory in order to apply predictable amount of forces.

The RS between bracket and wire depends mainly on classical friction (FR), in which the type of wire engaging system is influential, and binding (BI) [2]. FR is proportional to the normal force (FN), acting perpendicular to the direction of movement on the contact surface and depends on the coefficient of friction (*μ*) of a specific material according to the formula: FR = *μ* FN [3]. BI represents the force produced when the wire first contacts both opposing edges of the slot and is governed by the angular relationship between bracket slot and wire [4]. BI is encountered throughout the treatment, i.e., during arch alignment and leveling, space closure, or the finishing phase when the torque control is required for a correct tridimensional position of dental roots [5], and it is influenced by the wire stiffness [6]. Factors such as bracket type [7,8], type and method of ligation [9,10], bracket and arch-wire alloy [11,12], surface characteristics [11,13], wire-slot angulation [3,14,15], arch-wire size [15,16], and section [16,17] were found to affect the RS.

To the best of our knowledge, there are no studies evaluating how specific characteristics of slot design, such as the mesio-distal slot width and slot depth, influence the RS.

The aim of this study was to evaluate the effect of the abovementioned slot design characteristics on the RS recorded testing five different low-friction brackets.

## Methods

Four different types of self-ligating brackets and one conventional ligating bracket were tested (Table 1): Damon SL II (Ormco, Orange, CA, USA), In-Ovation (GAC International, Bohemia, NY, USA), Quick (Forestadent, Pforzheim, Germany), Time 2 *(*AO, Sheboygan, WI, USA), and Synergy (RMO, Denver, CO, USA). The Synergy bracket was tested in passive ligation configuration (i.e., with the ligature applied only to the central wings). All the tested brackets were first premolar brackets presenting the same vertical nominal dimension (0.022 in) and prescription (MBT system). The wires chosen for this study were supplied in straight lengths (Table 1); they were all 0.014-in NiTi thermal wires with nominal austenitic finish temperature stabilization at 36°C (Therma-Lite, AO). The ligatures used for the low-friction configuration of Synergy brackets (Table 1) were elastomeric modules (internal diameter 1 mm, Leone S.p.A*.*, Florence, Italy).

**Table 1:** Description of the materials used in this study

**Material**	**Characteristics**	**Manufacturer**
Brackets		
Damon SL II	Passive SLS	Ormco, Orange, CA, USA
	Slot 0.022 × 0.028 in	
	MBT prescription	
In-Ovation	Interactive SLS	GAC International, Bohemia, NY, USA
	Slot 0.022 × 0.028 in	
	MBT prescription	
Quick	SLS	Forestadent, Pforzheim, Germany
	Slot 0.022 × 0.028 in	
	MBT prescription	
Synergy	Conventional LS	RMO, Denver, CO, USA
	Slot 0.022 × 0.028 in	
	MBT prescription	
Time 2	Interactive SLS	AO, Sheboygan, WI, USA
	Slot 0.022 × 0.028 in	
	MBT prescription	
Wires		
Therma-Lite	0.014-in NiTi Thermal	AO, Sheboygan, WI, USA
Ligatures		
Elastomeric modules	Internal diameter 1 mm	Leone S.p.A., Florence, Italy

### Experimental apparatus

**Figure 1:**
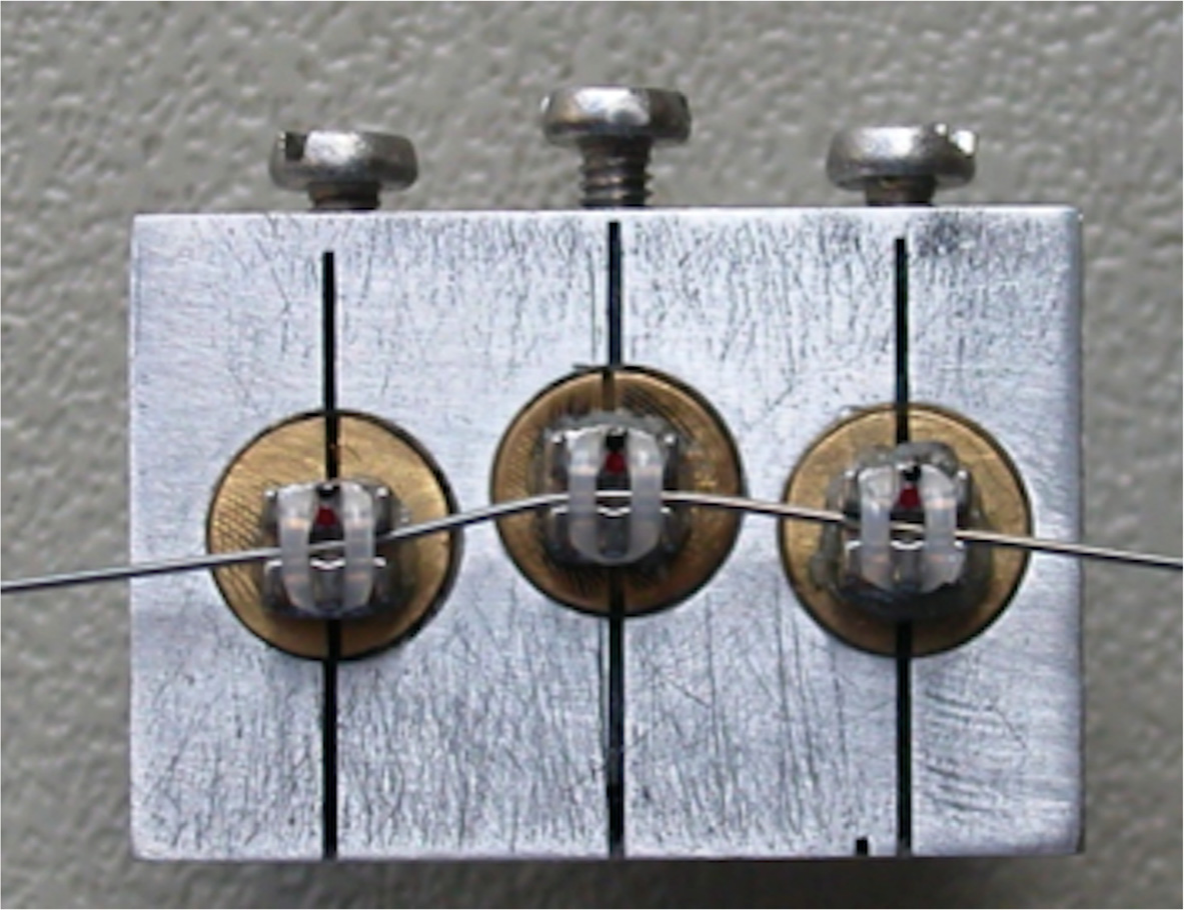
**Stainless steel support of the vertical bracket displacement experimental model.** The central bracket is 1 mm apically displaced relative to the adjacent brackets.

**Figure 2:**
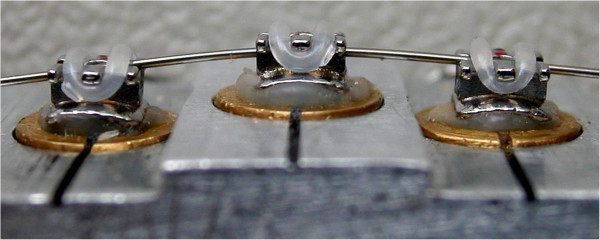
**Stainless steel support of the horizontal bracket displacement experimental model.** The central bracket is 1 mm buccally displaced relative to the adjacent brackets.

Vertical and horizontal bracket displacements were simulated with two different *in vitro* experimental models constituted by stainless steel supports holding three nonaligned brackets. The first experimental setting featured the central bracket 1 mm more apical compared to the other two adjacent brackets (Figure 1), while the second featured the central bracket 1 mm buccally displaced (Figure 2). The interbracket distance calculated from the center of each bracket was set at 8 mm as performed in a similar setting. [18] Both the experimental models were designed to evaluate the frictional behavior of the wire in the active configuration attending to the binding forces. The procedures of placement and bonding of the brackets are described in a previous studies [19-21].

### Testing machine

Static and kinetic frictions expressed by the 0.014-in NiTi thermal wires were measured by means of a customized testing machine based on the universal testing machine model. Details of the testing machine are reported in a previous study [19]. The machine recorded the average sum of the static friction in newton (N), calculated at the beginning of the test, and the kinetic friction (N) calculated during the test over about 100 data points of the first run of the wire throughout the set of brackets on a 5-mm piece of the orthodontic wire. One test was accomplished for each trio of brackets and each wire. All tests were repeated ten times, placing a new wire and a new trio of brackets at the end of each test. A thermostated room was used to keep the temperature at a constant value of 35.5°C [22] in a dry state during tests.

### Bracket design evaluation

**Figure 3:**
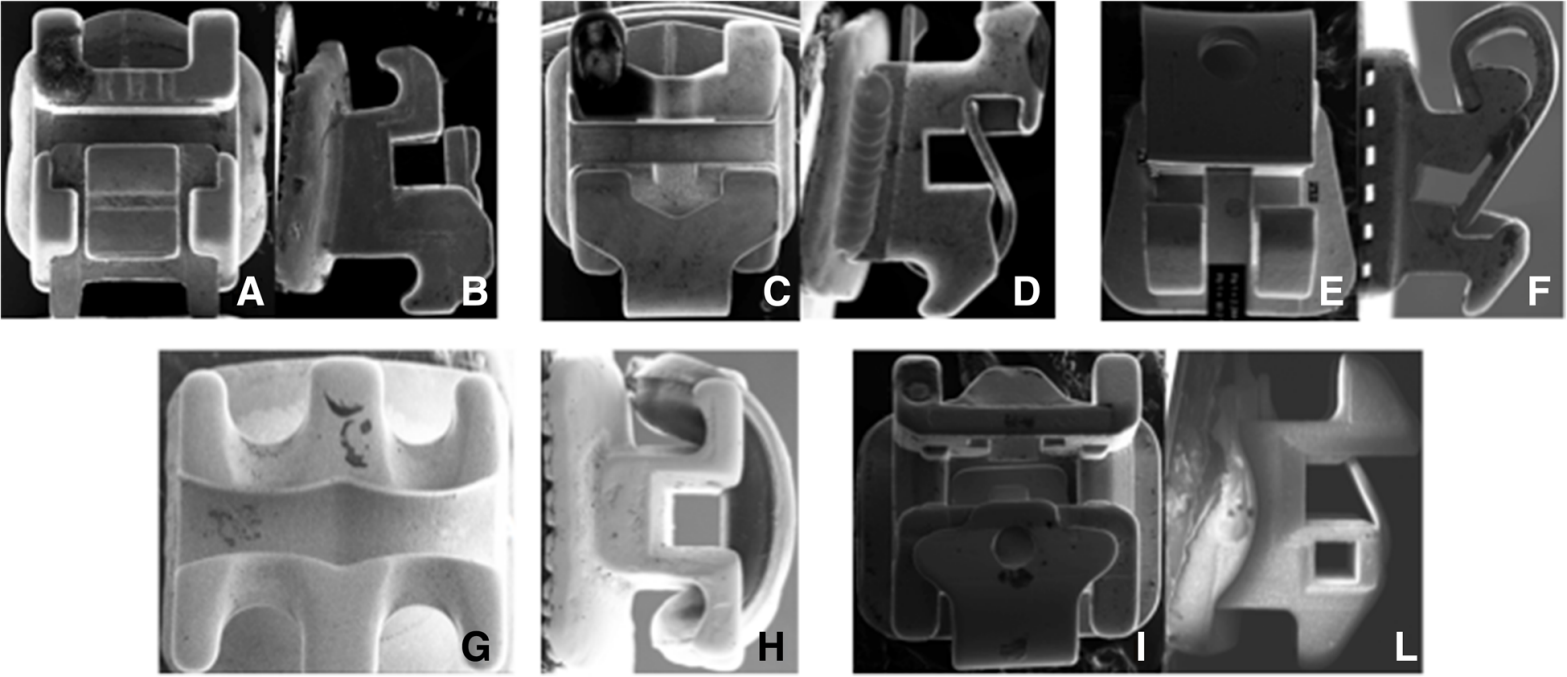
**Frontal and lateral views of scanning electron micrograph (SEM) images of tested brackets.** Damon SL II **(A**, **B)**, In-Ovation **(C**, **D)**, Time 2 **(E**, **F)**, Synergy **(G**, **H)**, and Quick **(I**, **L)**.

A scanning electron micrograph (SEM) (SUPRA, Carl Zeiss, Oberkochen, Germany) was used to take frontal and lateral pictures of all the tested brackets (Figure 3).

**Figure 4:**
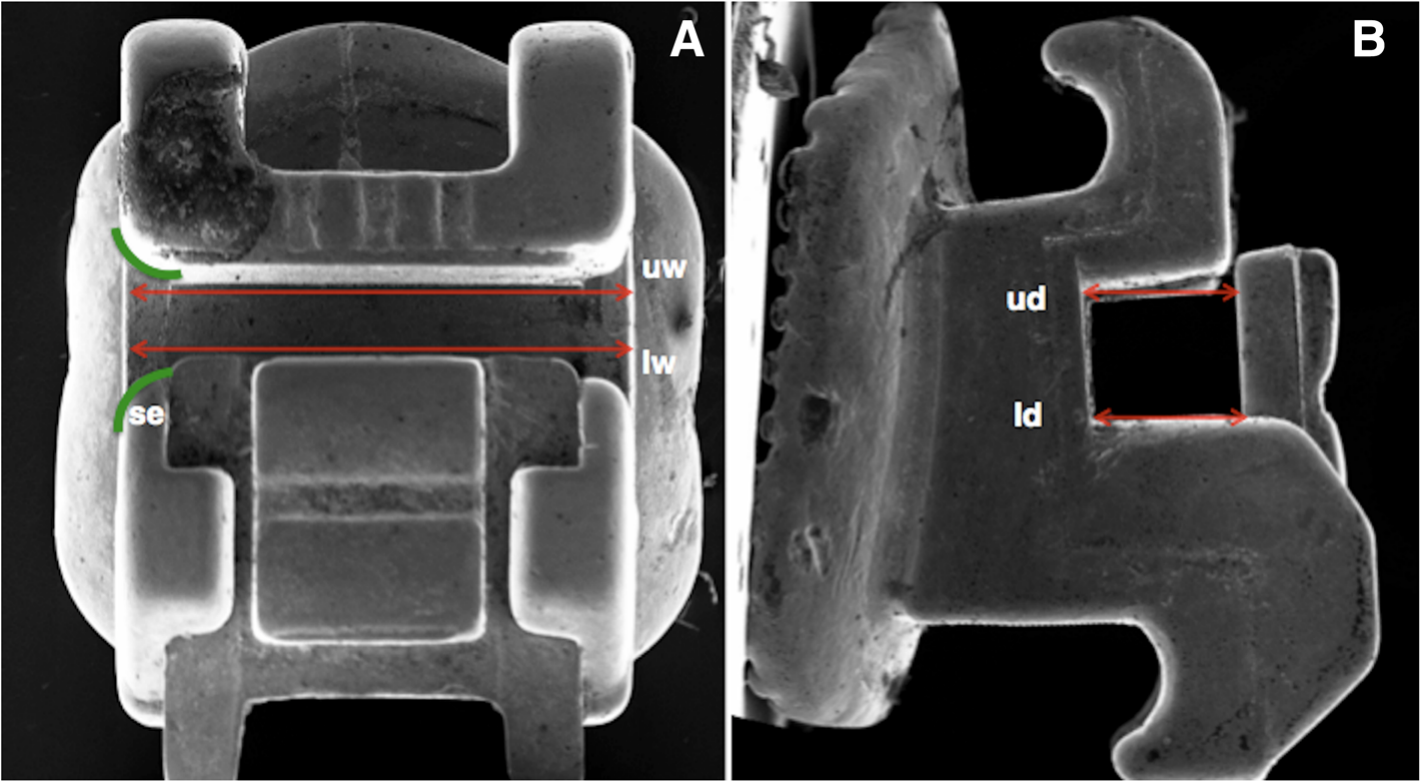
**Frontal (A) and lateral (B) views of scanning electron micrograph (SEM) images of Damon bracket.** Example of the measurements of slot design performed on Damon SL II bracket: upper width, uw; lower width, lw; upper depth, ud; lower depth, ld; and shape of the slot edges, se.

The evaluation of the design characteristics of each bracket slot was performed in investigating the following parameters: mesio-distal slot width, slot depth, slot height, and the presence of chamfered edges at the extremities of the slot. Five measurements of the upper and lower mesio-distal slot width, five measurements of the upper and lower slot depth, and five measurements of the slot height were taken for each bracket (Figure 4), and the mean value was calculated (Table 2). The same measurements were performed 1 month later, and the intraclass correlation coefficient (ICC) was used to evaluate the reliability between the first and second measurements; the ICC reported values ranged from 0.95 to 0.98 showing a systematic error adequate for an appropriate reproducibility of the measurements.

**Table 2:** Characteristics of slot design of each tested bracket

**Brackets**	**Slot width (mm)**	**Slot depth (mm)**	**Slot edges**
Damon SL II	2.7	0.7	chamfered
In-Ovation	2.9	0.56	chamfered
Quick	2.48	0.67	chamfered
Synergy	2.85	0.5	chamfered
Time 2	2.25	0.65	clear angle

### Statistics

Descriptive statistic of the frictional forces for both the experimental models was accomplished including mean, standard deviation, median, and minimum and maximum values (Tables 3 and 4). Before performing the inferential statistics, data set was analyzed using the Levene's Test for Equality of Variances. The one-way analysis of variance (ANOVA) and Student-Newman-Keuls tests were performed to compare the values of RS among the brackets tested in the vertical displacement experimental model. The levels of significance were set respectively at *p* < 0.001 and *p* < 0.05 for the two analyses (Table 3). The Kruskal-Wallis and Mann-Whitney with Bonferoni correction tests were accomplished for the comparison of RS values in the horizontal displacement experimental model. The levels of significance were set respectively at *p* < 0.001 and *p* < 0.05 for the two analyses (Table 4). Analysis of data was performed using MedCalc (MedCalc Software ver. 12.2.1.0, Mariakerke, Belgium) software.

**Table 3:** Descriptive and inferential statistics relative to the data obtained from the vertical bracket displacement model

**Vertical displacement**						
**Brackets**	**Number of tests**	**Mean**	**SD**	**Minimum**	**Maximum**	**Pairwise comparison of tensile bonding strengths**
Damon SL II	10	0.72	0.13	0.55	0.9	2, 4, 5
In-Ovation	10	1.1	0.17	0.89	1.4	1, 3, 4, 5
Quick	10	0.66	0.2	0.4	1.02	2, 4, 5
Synergy	10	0.92	0.06	0.83	1.05	1, 2, 3, 5
Time 2	10	1.48	0.12	1.23	1.63	1, 2, 3, 4

One-way ANOVA (*p* < 0.001) and Student-Newman-Keuls test (*p* < 0.05) for all pairwise comparison of data of resistance to slides (Newton) recorded among the tested brackets.

**Table 4:** Descriptive and inferential statistics relative to the data obtained from the vertical brackets displacement model

**Horizontal displacement**						
**Brackets**	**Number of tests**	**Mean**	**SD**	**Minimum**	**Maximum**	**Mann-Whitney test (Bonferroni's correction)**
Damon SL II	10	0.82	0.07	0.71	0.95	2, 4, 5
In-Ovation	10	1.01	0.1	0.78	1.13	1, 3, 4, 5
Quick	10	0.73	0.11	0.55	0.9	2, 5
Synergy	10	0.68	0.14	0.45	0.89	1, 2, 5
Time 2	10	1.35	0.08	1.27	1.53	1, 2, 3, 4

Kruskal-Wallis test (*p* < 0.001) and Mann-Whitney test with Bonferroni correction (*p* < 0.05) for comparison of data of resistance to slide (Newton) recorded among the five tested brackets.

To clarify the influence of the slot design on the RS recorded with both the experimental settings, the Spearman's rank correlation coefficient between mesio-distal slot dimensions and RS values and between slot depth dimensions and RS values was accomplished (Tables 5 and 6). Moreover, for each experimental model, the Spearman's rank correlation coefficient was repeated 5 times excluding one different bracket at each repetition (Tables 5 and 6). The data of mesio-distal and depth slot dimensions were preliminary evaluated with D'Agostino-Pearson normality test.

**Table 5:** Correlation between resistance to sliding and bracket slot dimension relative to vertical displacement setting

		**Bracket depth slot dimension**			**Bracket width slot dimension**		
**Number of evaluated brackets**	**Type of bracket**	***r***	***P *** **value**	**Significance**	***r***	***P *** **value**	**Significance**
5	(1), (2), (3), (4), (5)	-0.5	0.225	ns	0.0	0.525	ns
4	(2), (3), (4), (5)	-0.2	0.458	ns	-0.2	0.458	ns
4	(1), (3), (4), (5)	-0.6	0.208	ns	-0.2	0.458	ns
4	(1), (2), (4), (5)	-0.2	0.458	ns	-0.2	0.458	ns
4	(1), (2), (3), (5)	-0.6	0.208	ns	-0.2	0.458	ns
4	(1), (2), (3), (4)	-0.6	0.208	ns	1.0	0.042	*

Spearman's rank correlation coefficient. Damon SL II (1), In-Ovation (2), Quick (3), Synergy (4), Time 2 (5), *p < 0.05; not significant, ns.

**Table 6:** Correlation between resistance to sliding and bracket slot dimension relative to horizontal displacement setting

		**Bracket depth slot dimension**			**Bracket width slot dimension**		
**Number of evaluated brackets**	**Type of bracket**	***r***	***P *** **value**	**Significance**	***r***	**P value**	**Significance**
5	(1), (2), (3), (4), (5)	0.2	0.391	ns	-0.3	0.341	ns
4	(2), (3), (4), (5)	0.4	0.375	ns	-0.4	0.375	ns
4	(1), (3), (4), (5)	0.4	0.375	ns	-0.8	0.166	ns
4	(1), (2), (4), (5)	0.4	0.375	ns	-0.4	0.375	ns
4	(1), (2), (3), (5)	-0.6	0.208	ns	-0.2	0.458	ns
4	(1), (2), (3), (4)	0.4	0.375	ns	0.4	0.375	ns

Spearman's rank correlation coefficient. Damon SL II (1), In-Ovation (2), Quick (3), Synergy (4), Time 2 (5), *p < 0.05; not significant, ns.

## Results

The brackets tested can be listed in decreasing order of mesio-distal width and depth of the slot, respectively: In-Ovation, Damon II, Quick, Time 2, and Synergy; and Synergy, Quick, Time 2, Damon II, and In-Ovation (Table 2).

### Vertical displacement setting

The values of RS strongly differed (*p* < 0.001) among the brackets tested in this study, as revealed by the ANOVA. Time 2 and Quick showed, respectively, the higher (1.50 N, *p* < 0.05) and lower (0.66 N, *p* < 0.05) RS compared with the other tested brackets.

The In-Ovation produced higher frictional forces when compared with Quick, Synergy, and Damon (*p* < 0.05), while no significant differences were found between Damon II and Quick (*p* > 0.05) (Table 3).

A significant positive correlation (*p* = 0.042) was found between mesio-distal slot dimension and frictional values recorded when Time 2 was excluded; no significant correlation was found between slot depth dimensions and the values of RS registered (Table 5).

### Horizontal displacement

The values of RS strongly differed (*p* < 0.001) among the brackets tested in this study, as revealed by the ANOVA. Time 2 and Synergy showed, respectively, the higher (1.35 N, *p* < 0.05) and lower (0.68 N, *p* < 0.05) RS compared with the other tested brackets. No significant differences were found between Synergy and Quick and between Quick and Damon II (*p* > 0.05) (Table 4).

No significant correlation was found between the recorded values of RS and both the mesio-distal and depth slot dimensions (Table 6).

## Discussion

In the straight-wire biomechanics, the biological response of the orthodontic movement is limited by the friction generated during the sliding of the wire [23]. Studies have shown that approximately 50% of the force applied to reach the orthodontic dental movement is spent to overcome the frictional resistances [24] causing an inconsistent application of forces on the tooth. Minimizing the causes of RS may both speed tooth movement and increase its predictability.

In this study, we analyzed how the different slot designs of five low-friction brackets could affect the RS in two different experimental models (i.e., vertical and horizontal bracket displacements). The brackets chosen for this study were all reduced friction brackets; testing these brackets represents a great advantage because it is possible to reduce the experimental error constituted by the classical friction when a study aims to investigate the mechanical conditions governed by the binding.

Previous reports demonstrated that an increase of mesio-distal slot width, during vertical displacement of brackets, affects the BI by (a) reducing the interbracket distance, which increases the stiffness of the wire and (b) reducing the contact angle (*θ*c) [4]. Both of these phenomena increase the forces of binding and consequently the RS. In this study, we found a significant positive correlation between the mesio-distal slot width and frictional values when Time 2 bracket was excluded (Table 5). This finding suggests that wider mesio-distal slot dimensions cause higher frictional forces within brackets that are vertically displaced. According to these findings, clinicians should select brackets presenting a reduced mesio-distal width in order to minimize the RS.

The Time 2 bracket did not comply with this correlation; although it featured a reduced mesio-distal width, it showed higher levels of RS (1.50 N) compared with the other tested brackets (Table 3). The clear angle at the mesial and distal slot edges of Time 2, which is a specific feature of this bracket, could be responsible for this finding. Indeed, when the wire contacts both opposing edges of the slot (active configuration), the presence of clear edges could increase the binding of the wire and consequently the RS [25]. Therefore, bracket slot design featuring chamfered slot edges (Figure 4) could be an important requisite for a low-friction bracket system. The undersized vertical slot dimension of Time 2 (Table 2) could also contribute significantly to this finding.

The Quick bracket showed lower values of RS among the low-friction brackets tested in this setting. The oversized vertical slot dimension of this bracket (Table 2) along with the presence of chamfered slot edges could explain this result.

In horizontal bracket displacement, no significant correlation was found between RS and both slot depth and mesio-distal slot dimensions (Table 6); this result could be explained considering that the binding forces are influenced simultaneously by the abovementioned variables of slot design. These two variables affect each other, vary in an unrelated way among the brackets tested, and could explain the absence of any significant correlation.

The Quick bracket was found to express the lower values of RS among the evaluated self-ligating brackets despite that it did not feature the wider slot depth; it presented, instead, the shorter mesio-distal slot width, which allowed to a greater clearance of the wire within the horizontally displaced brackets.

In self-ligating brackets, the slot depth is also related to the type of wire engaging system [26,27] such as active, interactive, and passive that can influence the RS expressed when the brackets are horizontally displaced. In this study, the In-Ovation bracket, which featured a sloped clip, showed higher values of RS compared to the Damon II, which is a passive self-ligating bracket (Table 4). Our finding is in agreement with Kim et al. [28] who reported that the interaction wire/clip of active self-ligating brackets was responsible for higher values of RS found in comparison with passive self-ligating brackets even when the degree of malocclusion increased.

The Synergy bracket showed, in absolute, the lesser values of RS (0.68 N). This could be attributed to the behavior of the elastomeric module which caused an augmentation of the critical angle (*θ*c) by accommodating the deflection of the wire (Figure 2).

On the contrary, Time 2 was found to express the higher values of RS (1.35 N) despite that it featured a reduced mesio-distal slot width (Table 2); this bracket was the only one that did not present chamfered edges at the extremities of the clip and the base of the slot. This characteristic could be responsible for the higher binding between the wire and the clip itself, increasing the RS values, as suggested by other authors [23].

*In vitro* studies are reported to be unable to simulate exactly the *in vivo* conditions; [29,30] however, the *in vitro* settings are fundamental for the qualitative assessment of the singular variable that affect the overall resistance to slide.

## Conclusion

The bracket slot design is a fundamental aspect affecting the RS generated at the wire/bracket interface.

In the presence of brackets vertically displaced, the RS is directly influenced by the mesio-distal slot width. Furthermore, the absence of chamfered edges of the slot contributes to increase the RS.

In order to select the proper low-friction bracket system, clinicians should consider some characteristics of slot design such as mesio-distal slot dimension and the presence of chamfered slot edges of the bracket slot.

## Competing interests

The authors declare that they have no competing interests.

## Authors' contributions

RN has revised the manuscript for important intellectual contents. ALG has written the manuscript and performed the tests. GM has contributed to the design of the study. AA has taken the SEM images of the brackets and made the measurements for each brackets. EB has performed the analysis of data. PC has contributed to the design of the study. GC has contributed to the conception of the study and given final approval of the version to be published. All authors read and approved the final manuscript.
